# Medical device regulatory challenges in the UK are affecting innovation and its potential benefits

**DOI:** 10.1177/09544119231203776

**Published:** 2023-10-15

**Authors:** Jacqueline Beddoe-Rosendo, Clare L Heaysman, Joseph V Hajnal, Sebastien Ourselin, Anne Vanhoestenberghe

**Affiliations:** School of Biomedical Engineering and Imaging Sciences, King’s College London, London, UK

**Keywords:** Medical device regulation, MHRA, approved body capacity, innovation, UKCA marking

## Abstract

The increase in regulatory challenges on medical technology developed and deployed in the UK is having a negative impact on innovation. In this paper we show how the limited capacity of Approved and Notified Bodies is one more barrier in the innovation pipeline, that could push more teams to consider applying for FDA approval instead of UKCA marking, potentially limiting how much our patients benefit from the world-leading research undertaken in UK universities.

## Introduction

Historically there have been many challenges associated with translating medical technology developed in academia to the clinic, including poor understanding of regulations and lack of centralised support within academic institutions.^1–4^ However, a lot has been done in recent years to address the translational gap and increase the impact of technology, with many universities investing in quality management and regulatory expertise to assist with this process.^5–8^

At King’s College London, with support from the Wellcome Trust and UKRI, infrastructure has been steadily put in place to support medical device development projects. This includes the creation of a Quality Management System (QMS), which provides a framework for devices to be developed in line with the relevant regulations; support for a Quality Team, which runs the QMS and provides regulatory support; and the MAISI facility, a cleanroom dedicated to the manufacture of medical devices for first-in-human studies. These services sit in the wider ecosystem of the London Institute of Healthcare Engineering (LIHE) which aims to translate MedTech research rapidly into new products and technologies that will benefit patients.^9^

Despite these improvements, we find that the tightening regulations and their consequences on the capacity of EU Notified Bodies and UK Approved Bodies, which audit manufacturers against regulations, are a growing issue which is already impacting new device development. European Union medical device regulations have become more stringent in recent years,^10^ and, after further postponement, new regulations in the United Kingdom are expected to follow suit in 2025.^11^ At the same time, as a consequence of the increased burden imposed by these regulatory changes and Brexit, the number of Notified Bodies in the EU and Approved Bodies in the UK has decreased.^12–14^

This article aims to highlight some of the issues faced by medical device developers in the UK and encourage the government to take swift action to ensure the continued supply of innovative medical devices to support patients across the country.

Although the Asian Pacific market has grown significantly in recent years,^15^ this article focuses on the UK, EU, and USA due to their importance for UK-based medical device manufacturers for market entry.

## Case study

To illustrate some of the challenges faced by academic medical technology development projects, and their consequences on innovation, we discuss a virtual reality surgical planning tool which is currently under development at King’s. This device is intended to be used by clinicians to visualise CT, MRI and ultrasound images in 3D to allow for pre-operative analysis of surgical options.

The goals of this project include conducting a prospective study and developing a minimum viable system for commercialisation. Based on the intended use, the device was a Class I with measuring function in the UK, in accordance with the UK Medical Devices Regulations (2002) which transposes EU Directive 93/42/EEC on Medical Devices (MDD) into UK law.

For this device, the team considered applying for UK Conformity Assessed (UKCA) marking through equivalence to obtain market approval for the device in the UK before conducting the prospective study. Equivalence is the use of clinical data of another device that has the same or similar technical, biological and clinical characteristics to support the conformity assessment.^16^ By first obtaining the UKCA mark, any studies with the device would not require the UK’s Medicines and Healthcare products Regulatory Agency (MHRA) notification which is costly and time-consuming (Note: As of 2023, the MHRA assessment period is 60 days^17^ and initial notification of a clinical investigation costs have risen to £7472 with any subsequent amendments costing £207^18^). As a Class I medical device with measuring function, to obtain the UKCA mark, Approved Body involvement would only be required to review the ‘aspects of manufacture concerned with the conformity of the products with the metrological requirements’,^19^ which is meant to be a much quicker review compared with higher class devices that typically require full Quality Management System and Technical File review.

Unfortunately, due to the limited Approved Body capacity, the team had to change their plans. Currently there are only four Approved Bodies in the UK for medical devices, including invitro medical devices (IVDs). However, only three include in their scope this type of device. When they were contacted, none were available to work with new customers. One of the Approved Bodies stated that it would take at least 6 months to know if they would be able to take on the project and, in the case that they could, there would still be an additional wait for the review.

Academic research funding, like most R&D, is limited in time, without timely progress, the projects cannot continue. Such delays with the Approved Body make it impossible to obtain UKCA marking in time to conduct a prospective study, evaluating the device’s potential ahead of further grant applications to further clinical translation.

Consequently, the team adopted an alternative strategy and simplified the device by removing the measuring function. As a Class I medical device without measuring function, Approved Body involvement would not be required for UKCA marking. However, the device now has a reduced value proposition and does not offer the same level of potential benefits to the clinicians and patients.

Regrettably, the cost to deliver the originally intended device has significantly increased, as further funding will be required to support the much longer approval timeline for the device with the measuring function. This has led the team to consider bypassing the UK, and EU, markets entirely and applying initially for FDA clearance to obtain market access to the United States. This will be to the detriment of UK researchers, businesses, and patients.

## General trends

There is also a more general concern over the availability of medical devices in the EU and UK in the future. Clinicians are already noticing a reduction in the medical devices available for clinical use^20^ and the situation is only expected to get worse. The changes in regulations have led many manufacturers to drop product lines,^21^ which has contributed to the value of medical technology exports in the UK in 2022 dropping substantially compared to previous years.^22^ Additionally, the results from a recent survey conducted by Team NB, the European Association for Medical Devices of Notified Bodies, estimate that 6300 certificates can be issued per year but over 14,000 certificates were set to expire in 2024.^23^

Notified Body capacity is also affecting the time frames to obtain certification. Although an in-depth study of costs and timelines is beyond the scope of this paper, data obtained by consultancy company OpenRegulatory indicates that many Notified Bodies are not taking on any new clients or directly not answering emails.^24^

These organisations are struggling under the weight of the new regulations and the administrative burden is only set to increase due to the shortage gaming and the bullwhip effect^25^ as many regulatory experts are now recommending that manufacturers apply to several bodies at once in the hope that at least one of these organisations will take them on as a customer.

With the ongoing chaos in the United Kingdom and capacity limitations in the European Union, it is no surprise that many medical device developers are now looking to the United States.^26^ The US has been an attractive market for a long time as it is the single largest medical device market, accounting for 43.5% of the global market share.^27^ Although Europe is the second largest market, with 27.3% of the world market, the UK only accounts for 10.4% of this value (i.e. 2.84% of the total).^27^

Not only do FDA market approvals typically offer access to a much larger market in relation to the regulatory costs, the time frame for obtaining market approval is now significantly shorter. This has not always been the case, 20 years ago the EU was seen for some types of devices as offering a faster route to market.^28^ The FDA has since modernised its process, whilst the EU and UK are suffering from limited Notified Body and Approved Body capacity. The 510(k) pre-market notification route, which is the most common regulatory pathway for bringing medical devices to the US market,^29^ has a 90-day turnaround time,^30^ with criteria for equivalence generally considered more straightforward compared with EU medical device requirements.^31^ And the FDA has recently set timely goals for other approval pathways.^32^

The FDA is also putting effort into supporting devices to market. Through Q-submissions manufacturers can request a meeting with the FDA to obtain feedback prior to an intended premarket submission^33^ while Notified Bodies and Approved Bodies are expressly forbidden from doing so.^34^ The MHRA’s Innovation Office is available to answer questions from manufacturers, but, unlike the FDA, the remit of this service is limited to novel medicines, medical devices and methods.^35^ For other devices, it appears that the MHRA offers regulatory advice for a fee.^18^ Unsurprisingly, innovators and regulatory experts in the UK are recommending changes, for the MHRA to engage in constructive dialogue with device developers ahead of their submission, to minimise the burden on both sides.^36^

Additionally, for more innovative devices the FDA is exploring alternative routes to market, its recently published review of the software pre-certification pilot programme showed promising results.^37^

## The response from EU and UK regulators

A controversial position paper, published by the EU’s Medical Device Coordination Group (MDCG 2022-11^38^), suggested that manufacturers of devices lacked preparedness for the EU Medical Device Regulations and needed to apply to the Notified Bodies as soon as possible to avoid shortages of medical devices, without recognising the bottleneck that Notified Bodies have become. Tellingly, after pressure from industry and healthcare professionals, they have published a position paper (MDCG 2022-14^39^) highlighting the capacity issue and suggesting actions to help remedy the situation. Additionally, they have further extended the transition period for the EU Medical Device Regulations^40^ and issued guidance to allow legacy devices to be placed on the market with an expired CE certificate under Article 59 and Article 97.^41,42^ It is still to be seen whether these measures will be effective or if they are solely kicking the can down the road.

Although Notified Bodies generally agree with the measures introduced in MDCG 2022-14, a year on, several of the critical deliverables identified in this document are still pending.^43^ Further, additional measures are required to ensure sufficient Notified Body capacity, including shorter timelines for designation and the need for a stable regulatory environment.^43^

The MHRA’s consultation on the future of the medical device regulations in the UK also highlights the widespread concern in the industry, and a desire to see the government take decisive actions to address the issue in a timely manner. Indeed, to ensure that the UK remains an attractive market and the continued supply of medical devices, respondents were in favour of introducing alternative routes to market, such as accepting approvals from other countries, and the MHRA obtaining additional powers to grant initial market approval for innovative medical devices, which should facilitate technology transfer and adoption in the future.^44^ Since then, the MHRA with other partners has launched the Innovative Devices Access Pathway (IDAP) although few details were available at the time of publication.^45^

Although feedback from the consultation seems promising, new legislation is not expected until at least July 2025 and, if these improvements are included, there will be a transition period until these come into effect. Additionally, these suggestions would increase the workload of the MHRA and are coming after a period when budget cuts and redundancies^46^ significantly decreased the number of staff at the agency.^47^

While we wait for the new legislation, a recent amendment to the current legislation has extended the timeframes for allowing CE marked medical devices to be placed on the Great Britain market until 30th June 2028 if they were CE marked under the EU Medical Device Directive or EU Active Implantable Medical Device Directive, and 30th June 2030 if they were CE marked under the EU Medical Device Regulations.^48^ Due to the high level of uncertainty and the rapidly changing environment, we urge the reader to check the European Commission and MHRA websites for the latest updates.

## Conclusions

Uncertainty and changes in regulation present new challenges for the UK MedTech community, which are exacerbated by the limited Approved Body and Notified Body capacity, and limited support offered. This has already impacted innovation, as device developers look to the US for certification. Although regulators are finally trying to make changes to address these capacity issues, there is no clarity on what exactly will be implemented and whether this will improve the current situation.

As the MHRA works on new UK regulations, we implore them to consider the response to their consultation, listen to industry and Approved Bodies, learn from the mistakes made during the implementation of the EU regulations and carefully assess the impact that the new UK regulations will have on the availability of devices, with particular attention to timeframes and support available for those who wish to enter the UK market.

As we have left the EU single market, for the UK, now alone, to become an attractive place to launch truly transformative medical devices, we need to act fast to address the challenges within the innovation pipeline. For the sake of patients and the NHS, we need to ensure that regulators support innovation and that the UK remains an attractive market for manufacturers.
